# Phonon-assisted relaxation between triplet and singlet states in a self-assembled double quantum dot

**DOI:** 10.1038/s41598-021-94621-7

**Published:** 2021-07-27

**Authors:** Krzysztof Gawarecki, Paweł Machnikowski

**Affiliations:** Department of Theoretical Physics, Faculty of Fundamental Problems of Technology, Wrocław University of Science and Technology, Wybrzeże Wyspiańskiego 27, 50-370 Wrocław, Poland

**Keywords:** Spintronics, Quantum dots

## Abstract

We study theoretically phonon-induced spin dynamics of two electrons confined in a self-assembled double quantum dot. We calculate the transition rates and time evolution of occupations for the spin-triplet and spin-singlet states. We characterize the relative importance of various relaxation channels, including two-phonon processes, as a function of the electric and magnetic fields. The simulations are based on a model combining the eight-band $\varvec{k}\!\cdot \!\varvec{p}$ method and configuration-interaction approach. We show that the electron g-factor mismatch between the Zeeman doublets localized on different dots opens a relatively fast triplet-singlet phonon-assisted relaxation channel. We also demonstrate, that the relaxation near the triplet-singlet anticrossing is slowed down up to several orders of magnitude due to vanishing of some relaxation channels.

## Introduction

Isolated spins in solid-state systems offer fast optical control methods, long-time stability and high-fidelity conversion to photonic flying qubits^1–3^. One of the possible implementations of solid-state spin qubits are single electrons in quantum dots (QDs), which are optically active systems that offer the possibility of quantum coherent spin initialization, storage, readout, as well as entangling spins with photons^4–11^. Recent progress in controlling spin-photon coupling in these systems^2^ paves the way towards QD-based integrated photonic technologies^12^, while hyperfine interactions, one of the main sources of spin decoherence, can be controlled with increasing precision^13–15^.

A system composed of two coupled QDs (a quantum dot molecule, QDM) offers an additional degree of freedom related to carrier localization, which can be controlled by an external electric field^16,17^. In such systems, two electrons can be trapped in the ground-state manifold of the two dots, forming singlet and triplet states with inhomogeneous coherence times exceeding 200 ns^18^. The singlet-triplet state manifold in an optically active QDM gives rise to a specific structure of optical transitions that can be exploited in quantum-optical schemes^19,20^ that are of both fundamental and application-oriented interest. In particular, it offers both spin-selective, as well as non-selective optical couplings to four-particle configurations, which are essential for state preparation and readout^3,21,22^.

Both information storage and quantum state readout via light scattering rely on the stability of the spin configurations in the two-electron system. The relaxation rates between the triplet and singlet states were previously studied in gate-defined GaAs QDMs^23–25^ and Si/SiGe systems^26–28^. In the first case, the spin relaxation times on the order of hundreds of micro-seconds were predicted for relatively low barriers and large dots, growing by orders of magnitude when the barrier height increases or the QD size decreases^23^. It has been shown^29^, that a difference between the site-dependent g-tensors in a QDM couples singlet to triplet states changing the leakage current.

In self-assembled QDs, spin relaxation can be induced by many mechanisms resulting from band mixing and strain, out of which the shear-strain-induced spin-orbit coupling was shown to dominate the single-electron spin relaxation within the ground-state Zeeman doublet of a single QD^30^. The dependence of the strain, band-mixing, and spin-orbit effects on the QD geometry and composition profile requires precise modeling of carrier states and carrier-phonon couplings. Therefore, the methods and results relevant to gate-defined QDs are not directly transferable to self-assembled systems. Reliable and computationally cost-effective modeling of self-assembled systems is possible using $\varvec{k}\!\cdot \!\varvec{p}$ methods within the envelope function formalism^31^. This approach has been used to calculate spin-conserving relaxation between two-electron singlet states in self-assembled QDMs, yielding relaxation times on the order of tens of picoseconds^32^. Single-electron spin relaxation between Zeeman sub-levels, modeled using the eight-band $\varvec{k}\!\cdot \!\varvec{p}$ method, takes place on the time scales of $\sim 100$ ms at 1 Tesla, scaling as $B^{5}$ at low and moderate magnetic fields^30^.

In this work, we model theoretically phonon-induced relaxation between two-electron states in a self-assembled QDM. We calculate single-particle states using the 8-band $\varvec{k}\!\cdot \!\varvec{p}$ model with strain distribution found within continuous elasticity approach. The Coulomb coupling between the electrons is taken into account via configuration interaction (CI) method. Then the phonon-assisted transition rates are calculated using Fermi golden rule. We also model two-phonon processes within the time-convolutionless (TCL) projection operator method. We investigate the relaxation processes at various electric and magnetic fields, which can be used to control the system. We show that the difference between the *g*-factors in the two QDs, which naturally emerges due to their strain and geometrical characteristics, enhances the transitions from one of the triplet states, providing the dominant relaxation channel for a wide range of magnetic field. Thus, triplet–singlet spin relaxation in self-assembled structures is dominated by a real-space spin-orbit effect, which is relevant to transitions between states with the same *z* component of the spin but belonging to different representations of the rotation group and therefore affects only many-particle states. This relaxation is strongly suppressed at the electric field corresponding to the minimum singlet-triplet splitting (the “sweet spot”). We discuss also the kinetics of transition between the two-electron spin states and show that, depending on the magnetic field and temperature, various direct and sequential processes may contribute to the relaxation towards the singlet ground state. Finally, we show that the two-phonon transitions are important at the ”sweet spot” for temperatures from several Kelvins.

The paper is organized as follows. In section “Model”, we describe the model used to calculate the single and double electron states, as well as the phonon-assisted relaxation. In section “Results” we present the results of numerical simulations. Finally, section “Conclusions” contains concluding remarks.

## Model

**Figure 1:**
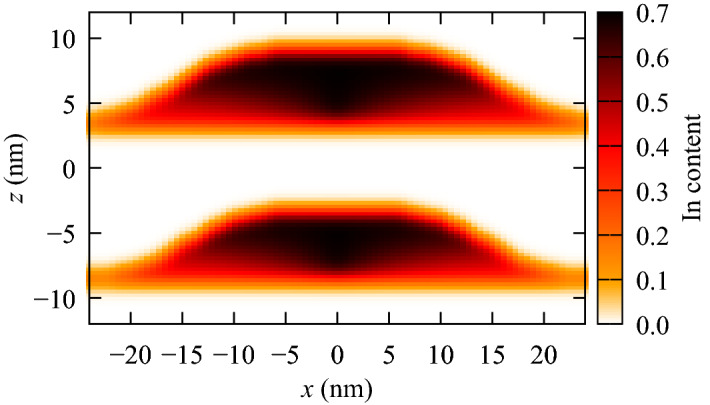
$\hbox {In}_x\hbox {Ga}_{1-x}$As distribution in the QDM system.

We consider two self-assembled, vertically stacked InAs/GaAs QDs^16^. The composition gradient in the dots is modeled as a trumpet shape^33^ with the maximum In content of 0.7 and the minimum of 0.4 (see Fig. 1). The mathematical models of the dot geometry and composition are described in Ref.^34^. The heights of the lower (“l”) and the upper (“u”) dot are taken $h_{\mathrm {l}} = 8.5 \, a_G$ and $h_{\mathrm {u}} = 9.5 \, a_G$ respectively, where $a_G = 0.565325$ nm is the GaAs lattice constant. The dots are placed on wetting layers of 0.4 In content and thickness of a single $a_G$. The parameters defining approximate QD base radii are chosen as $r_{\mathrm {l}}=28 \, a_G$ and $r_{\mathrm {u}}=30 \, a_G$. The material intermixing is accounted for via a Gaussian blur (where we took 0.6 nm of the standard deviation). The simulations are performed for the axially oriented magnetic (*B*) and electric (*F*) fields.

We calculate single-electron states $\left| \phi _n\right\rangle$ using the eight-band $\varvec{k}\!\cdot \!\varvec{p}$ model^35,36^. We take into account strain distribution^37^ and piezoelectric field^38,39^ in the system. We used computational box $200 a_G \times 200 a_G \times 200 a_G$ for strain simulations and $100 a_G \times 100 a_G \times 70 a_G$ for finding the single-particle states. The model and the details of its implementation are given in Ref.^40^, here we use an extended version, described in Ref.^41^. The electric field is incorporated in the Hamiltonian via the standard band-diagonal term

$$\begin{aligned} V^{({\mathrm {F}})}(z) = F (z - z_0), \end{aligned}$$

where $z_0$ is taken in the middle of the computational box. To preclude escaping the electron through the barrier in high electric fields, we take constant values: $V^{({\mathrm {F}})}_{\mathrm {l}} = V^{({\mathrm {F}})}(z_{\mathrm {l}})$ for $z < z_{\mathrm {l}}$ and $V^{({\mathrm {F}})}_{\mathrm {u}} = V^{({\mathrm {F}})}(z_{\mathrm {u}})$ for $z > z_{\mathrm {u}}$, where $z_{\mathrm {l}}$, $z_{\mathrm {u}}$ is the bottom of the lower dot (counted with the wetting layer) and the top of the upper one, respectively. Finally, we calculate the two-electron states

$$\begin{aligned} \left| \Psi _n\right\rangle = \sum _{ij} c^{(n)}_{ij} a^\dagger _i a^\dagger _j \left| {\mathrm {vac.}}\right\rangle , \end{aligned}$$

where $\left| {\mathrm {vac.}}\right\rangle$ is the crystal ground state, the coefficients $c^{(n)}_{ij}$ are found from exact diagonalization of the Coulomb interaction Hamiltonian within the CI approach, and $c^{(n)}_{ij}=0$ for $j \ge i$. The CI basis contains 4 lowest single-electron states (i.e. two spin-dependent *s*-type states in each dot). In an idealized case (no spin-orbit interaction and no tunnel coupling), one can obtain the well known singlet/triplet configurations

$$\begin{aligned} \left| S(0,2)\right\rangle&= a^\dagger _{{\mathrm {u}} \uparrow } a^\dagger _{{\mathrm {u}} \downarrow } \left| {\mathrm {vac.}}\right\rangle ,\\ \left| T_+(1,1)\right\rangle&= a^\dagger _{{\mathrm {u}} \uparrow } a^\dagger _{{\mathrm {l}} \uparrow } \left| {\mathrm {vac.}}\right\rangle ,\\ \left| T_0(1,1)\right\rangle&= \frac{1}{\sqrt{2}} \left[ a^\dagger _{{\mathrm {u}} \uparrow } a^\dagger _{{\mathrm {l}} \downarrow } - a^\dagger _{{\mathrm {l}} \uparrow } a^\dagger _{{\mathrm {u}} \downarrow } \right] \left| {\mathrm {vac.}}\right\rangle ,\\ \left| T_-(1,1)\right\rangle&= a^\dagger _{{\mathrm {u}} \downarrow } a^\dagger _{{\mathrm {l}} \downarrow } \left| {\mathrm {vac.}}\right\rangle ,\\ \left| S(1,1)\right\rangle&= \frac{1}{\sqrt{2}} \left[ a^\dagger _{{\mathrm {u}} \uparrow } a^\dagger _{{\mathrm {l}} \downarrow } + a^\dagger _{{\mathrm {l}} \uparrow } a^\dagger _{{\mathrm {u} }\downarrow } \right] \left| {\mathrm {vac.}}\right\rangle ,\\ \left| S(2,0)\right\rangle&= a^\dagger _{{\mathrm {l}} \uparrow } a^\dagger _{{\mathrm {l}} \downarrow } \left| {\mathrm {vac.}}\right\rangle , \end{aligned}$$

where $(N_{\mathrm {l}},N_{\mathrm {u}})$ describes the nominal occupations in the dots, $a^\dagger _{({\mathrm {l/u}}) (\uparrow / \downarrow ) }$ is the creation operator for the electron state in the lower/upper dot and $\uparrow / \downarrow \,$ denotes the spin orientation. Because of the SO coupling, spin is no longer a good quantum number. Furthermore, spatial configurations are mixed by the tunnel coupling. However, spin-mixing effects are generally small, and the above notation is useful to classify well localized states (far from the tunnel resonances).

The spin-related properties of many-particle states can be characterized by the $D_{j}$ irreducible representations of the full rotational group, where *j* is related to the total angular momentum^42,43^. In the case of a two electron system, the direct product of representations gives $D_{1/2} \otimes D_{1/2} = D_{0} + D_{1}$. In consequence, states can belong to the one-dimensional trivial representation $D_{0}$ (singlet states) or to the three-dimensional $D_{1}$ (threefold degenerated triplet states). In the presence of the SO coupling, the geometrical symmetry breaking affects also the spin degree of freedom. For the QDM considered here, at $B=0$ T the system is described by the $C_{2v}$ symmetry point group. In this group, $D_{1}$ splits into one- and two-dimensional representations^42,43^, which lifts the degeneracy of the $T_\pm$ and $T_0$ triplet states.

Spin-orbit interaction creates various channels for phonon-assisted spin-flip processes. One class of such effects is driven by *spin admixture* mechanisms, where the single-particle state with some nominal spin orientation gets a contribution of the opposite spin^30,44–46^. The other class contains direct spin-phonon coupling mechanisms^46–48^, which in QDs are typically weaker compared to the channels due to the spin admixture^30,44,45^.

The phonon-induced transition rates between the two-electron states can be calculated using the Fermi golden rule^49^

$$\begin{aligned} \Gamma (n \rightarrow m)&= \frac{2 \pi }{\hbar ^2} \left| n_{\mathrm {B}}(\omega _{mn}) + 1\right| \sum _{\varvec{k},\lambda } \left| G_{nm,\lambda }(\varvec{k})\right| ^2 [ \delta (\omega _{mn} - \omega _{k,\lambda }) + \delta (\omega _{mn} + \omega _{k,\lambda })], \end{aligned}$$

where $\omega _{mn} = (E_{n} - E_{m})/\hbar$ is related to the energy difference between the initial and final state, $n_{\mathrm {B}}(\omega )$ is the Bose–Einstein distribution, $\lambda$ denotes the phonon branch, and $\omega _{\varvec{k},\lambda } = c_{\lambda } k$ with branch-dependent speed of sound $c_{\lambda }$. Finally, for the two-electron states

$$\begin{aligned} G_{nm,\lambda }(\varvec{k}) =&\sum _{ii'jj'} c^{*(n)}_{ij} c^{(m)}_{i'j'} \Big [ F_{ii',\lambda }(\varvec{k}) \delta _{jj'} - F_{ij',\lambda }(\varvec{k}) \delta _{ji'} - F_{ji',\lambda }(\varvec{k}) \delta _{ij'} + F_{jj',\lambda }(\varvec{k}) \delta _{ii'} \Big ], \end{aligned}$$

where

$$\begin{aligned} F_{ij,\lambda }(\varvec{k}) = \langle {\phi _i}\vert{ H_{\mathrm {int}}(\varvec{k},\lambda ) e^{i \varvec{k}\varvec{r}}} \vert {\phi _j},\rangle \end{aligned}$$

where $H_{\mathrm {int}}(\varvec{k},\lambda )$ is the carrier-phonon interaction Hamiltonian for a single phonon mode. We take into account the deformation potential and piezoelectric electron-phonon couplings^50,51^. The detailed description of the model and carrier-phonon Hamiltonian is given in Ref.^41^.

## Results

In this Section we discuss the calculated phonon-assisted relaxation rates. We also present the simulations of quantum kinetics for the two-electron singlet and triplet states.

### Single-phonon spin relaxation rates

**Figure 2:**
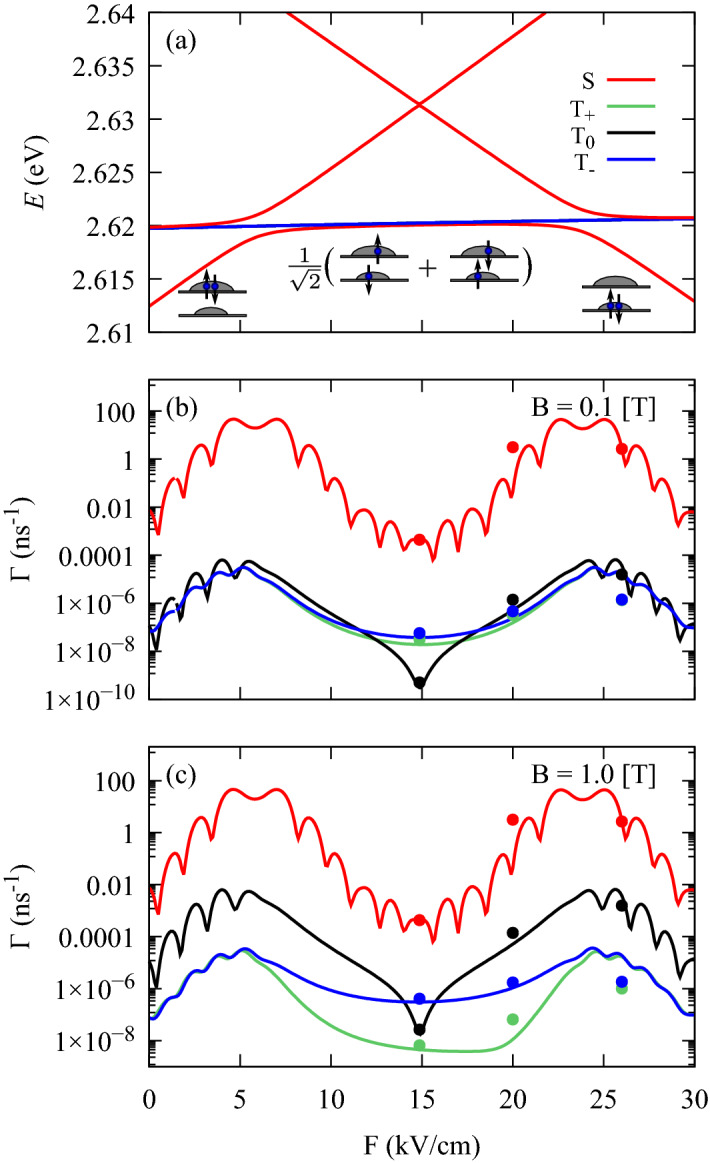
(**a**) Two-electron energy levels as a function of electric field. The sketches present (approximate) spins projections and occupations in the lowest singlet state. (**b**,**c**) Phonon-assisted transition rates to the lowest singlet state from: the first upper singlet (red solid line), triplet $T_+$ (green solid line), $T_0$ (black solid line), $T_-$ (blue dashed line). We assume $T=0$ K. The points in (**b**,**c**) correspond to CI calculations in the extended basis of 24 electron states.

The energy spectrum for the lowest two-electron states is shown in Fig. 2a. At $F=0$, the ground state has (approximately) a configuration of *S*(0, 2), where both electrons are localized in the upper dot (which is assumed to be the larger one). The next three energy levels are nominally triplet states $T_\pm \equiv T_\pm (1,1)$, and $T_0 \equiv T_0(1,1)$. At $B=0$, the energy splitting between them is up to several neV, hence can be neglected. The next state forms nominally the *S*(1, 1) configuration. Finally, the energetically highest state is *S*(2, 0).

For the nonzero electric field, we obtained the well known structure of energy branches with avoided crossings. Since the positive electric field decreases (increases) the energy in the lower (upper) dot, the states can be tuned into resonance. In consequence, at $F \approx 5.82$ kV/cm and $F \approx 23.84$ kV/cm, there are pronounced avoided crossings, corresponding to the tunneling of a single electron between the dots. The anticrossing at $F \approx 14.86$ kV/cm is very narrow, which is due to two-particle character of the involved tunneling process. Furthermore, at $F \approx 14.87$ kV/cm there is a “sweet spot”, which corresponds to minimal difference (approximately 0.19 meV) between the lowest singlet and triplet configurations.

First, we calculate the phonon-assisted relaxation rates between the two lowest singlet states (red lines in Fig. 2b,c). Depending on the magnitude of the electric field, the rates can describe different tunnel transitions: $S(1,1) \rightarrow S(0,2)$; $S(0,2) \rightarrow S(1,1)$; $S(2,0) \rightarrow S(1,1)$ or $S(1,1) \rightarrow S(2,0)$. The two pronounced maxima corresponding to the energy resonances from Fig. 2a are related to the increased overlap between the wavefunctions (which is due to spatial delocalization). The emission of longitudinal acoustic phonons from quantum dots preferentially takes place in the strongest confinement direction^52^, which in our case is the *z*-th axis. A phonon-assisted transition can be thought of as taking place at the one QD or the other. Constructive interference of quantum amplitudes for these two processes happens when the phonon wave is out of phase at these two points, hence, the phonon emission is expected to be enhanced for the frequencies $\omega = \pi c_l (2n + 1)/D$, where *n* has integer values and $c_l$ is the (longitudinal) sound velocity^32,53^. This is manifested by the oscillations of the transition rates^53,54^.

Since the transitions between the singlet states are spin conserving, they do not significantly depend on magnetic field. The rates on the order of tens of $\hbox {ns}^{-1}$ for the dots separated by about 10 nm are consistent with former predictions based on the single-band $\varvec{k}\!\cdot \!\varvec{p}$ approximation^32^.

Next, we calculate phonon-assisted transition rates from the triplet states to the lowest singlet state (Fig. 2b,c). Depending on the electric field, such processes can either involve tunneling [$T(1,1) \rightarrow S(0,2)$ and $T(1,1) \rightarrow S(2,0)$], or conserve QD occupations [$T(1,1) \rightarrow S(1,1)$], or take an intermediate form. For the inter-dot tunnel transitions the rates oscillate, while no oscillations appear for the charge conserving processes. In order to check the accuracy of our CI approach, we calculated the transition rates in an extended basis of 24 electron states. The results for $F=14.87$ kV/cm, $F = 20$ kV/cm, and $F = 26$ kV/cm are marked as points in Fig. 2b,c. At $F=14.87$ kV/cm (the sweet spot) the rates are up to 65% larger compared to the minimal basis of 4 states. This can be partially attributed to changes in the energy differences (in the extended basis the $T(1,1)-S(1,1)$ splittings are up to 13% larger). Also in the region of the inter-dot tunneling the rates are shifted, which is further enhanced by larger $T(1,1)-S(2,0)$ energy splittings (at $F = 26$ kV/cm these energy differences are up to 36% larger in the extended basis) and by possible changes in the localization of the electrons.

This is, however, a merely quantitative correction. Moreover, in our calculations, we cover rates spanning over many orders of magnitude. As extending the basis is demanding in the computational cost (in particular for the two-phonon processes), we perform further calculations in the original basis of the 4 states, keeping in mind that the actual values near the sweet spot may be larger.

The transitions $T_{\pm } \rightarrow S(0,2)$ and $T_{\pm } \rightarrow S(2,0)$ can be considered (approximately) as a single-electron tunneling accompanied by the spin-flip, while the other electron plays the role of a passive spectator. Such transitions are driven (primarily) by the spin admixture mechanism related to the Dresselhaus spin-orbit coupling^55^. Also the rates $\Gamma (T_\pm \rightarrow S(1,1) )$ corresponding to the charge conserving processes, at low magnetic field crucially depend on the Dresselhaus coupling.

The relaxation processes $T_{0} \rightarrow S(0,2)$ and $T_{0} \rightarrow S(2,0)$ exhibit different behavior compared to those involving the $T_{\pm }$. We have verified that, in this case, the spin admixture mechanisms described above play minor role. Instead, the dominant transition channel is related to the difference of the single-particle *g*-factors for the states localized on different dots. This is a real-space spin-orbital effect connecting the position to the spin degree of freedom. It can be interpreted with the effective Zeeman Hamiltonian

$$\begin{aligned} H_{\mathrm {Z}} = \frac{1}{2} \mu _B g_1 \sigma ^{(1)}_z B_z + \frac{1}{2} \mu _B g_2 \sigma ^{(2)}_z B_z, \end{aligned}$$

where $g_{1/2}$ and $\sigma ^{(1/2)}_z$ are *g*-factors and the (*z*-th) Pauli matrices defined for a given single-particle orbital (1 or 2). While such a Hamiltonian conserves the projection of the total angular momentum $J_z$, it does not commute with the $J^2$ operator, hence mixes the states belonging to different representations of the rotation group. This mixing effect was shown to be an important factor determining transport properties of a gate-defined double quantum dot^29^. Such an effect takes place only for many-body configurations. In the case of two electrons, the $T_\pm$ states are unaffected because $\langle {S}\vert{H_Z}\vert{T_\pm }\rangle =0$. On the other hand, $\langle {S(1,1)}\vert{H_Z}\vert{T_0}\rangle =\frac{1}{2} \mu _B (g_1 - g_2) B_z$ which mixes the states. This gives rise to a spin-mixing that leads to a phonon-assisted relaxation^30,44,45^. For the QDM system considered here, $g_{\mathrm {u}} \approx -1.05$ vs. $g_{\mathrm {l}} \approx -0.88$ for the upper and the lower dot respectively, opening a significant $T_0 \rightarrow S$ relaxation channel. In consequence, the transition rate $\Gamma (T_{0} \rightarrow S(1,1))$ shows a minimum at $F\approx 14.88$ kV/cm, which is near the point where the single-particle g-factors are equal [$g \approx (g_{\mathrm {u}} + g_{\mathrm {l}})/2$ ] due to the delocalization of the electron states. This is very close to, although not exactly coinciding with, the S-T “sweet spot” at $F \approx 14.87$ kV/cm.

**Figure 3:**
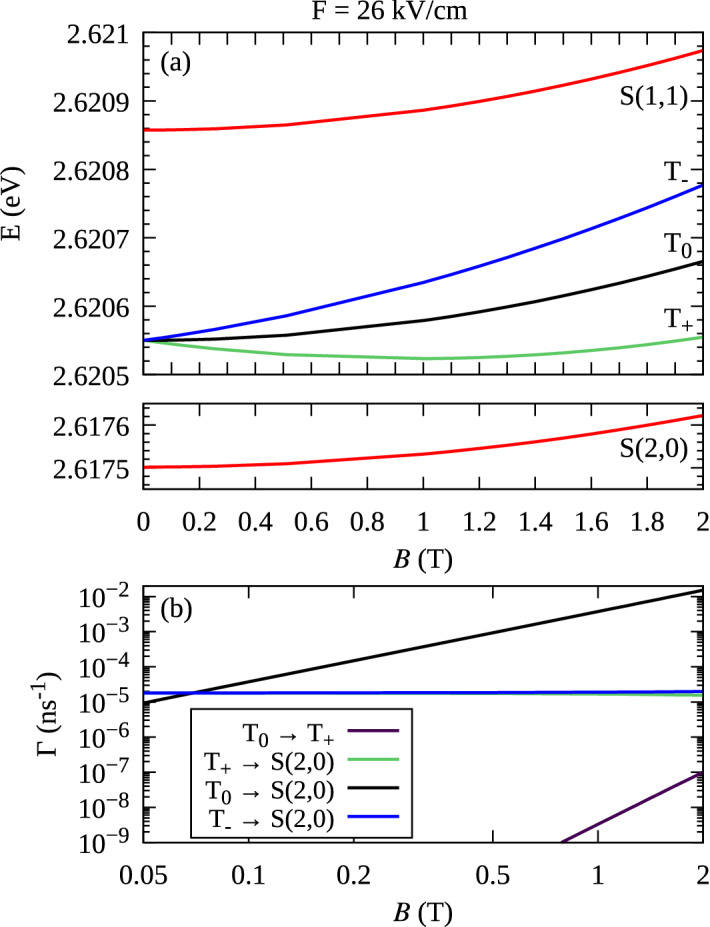
Energy branches (**a**) and phonon-assisted relaxation rates at $T=0$ K (**b**) as a function of axial magnetic field *B*, at $F=26$ kV/cm.

The magnetic-field dependence of the lowest two-electron states at $F=26$ kV/cm is shown in Fig. 3a. The energies of all the states contain a diamagnetic contribution $\propto B^2$. In addition, the energies of the spin-polarized states $T_\pm$ show a Zeeman shift. Since the single-electron g-factors are negative, the $T_{+}$ state has a lower energy than $T_{-}$. The calculated phonon-assisted relaxation rates between the states are shown in Fig. 3b. For the considered magnetic-field range, one can approximate the dependence $\Gamma (T_{\pm } \rightarrow S(2,0)) \approx \mp a B + c$. The dominant component *c* depends (mainly) on the Dresselhaus spin-admixture mechanism^55^. The relaxation from the $T_{0}$ state follows $\Gamma (T_{0} \rightarrow S(2,0)) \approx a_0 B^2$, where the parameter $a_0$ mainly depends on the difference between the single-particle g-factors of the Zeeman doublets $a_0 \propto (g_{\mathrm {u}}-g_{\mathrm {l}})^2$, consistent with the discussion in terms of the effective Zeeman Hamiltonian, presented above. Finally, the relaxation $T_0 \rightarrow T_+$ can be viewed as a single-electron spin-flip in the individual dots. The rate $\Gamma (T_0 \rightarrow T_+)$ depends on magnetic field as $\propto B^5$, which is consistent with the results for one electron in a single QD^30,44,45^.

**Figure 4:**
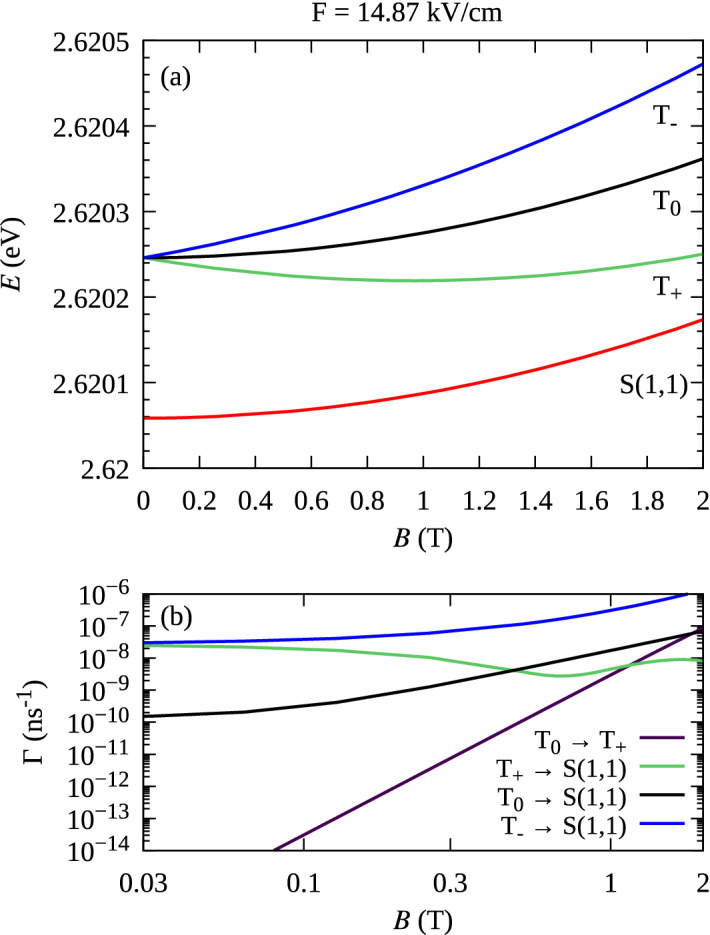
Energy branches (**a**) and phonon-assisted relaxation rates at $T=0$ K (**b**) as a function of axial magnetic field *B*, at the “sweet spot” $F=14.87$ kV/cm.

Next, we calculate the energy branches (Fig. 4a) and the relaxation rates (Fig. 4b) for the electric field corresponding to the sweet spot ($F=14.87$ kV/cm). The observed dependencies of the rates result from the interplay of various SO mechanisms (which can interfere constructively or destructively), and Zeeman energy splitting (important for the considered energy scale). We have checked that if the influence of the Zeeman splitting were (artificially) neglected, the rates could be very well fitted by the expression $\Gamma (T_\pm \rightarrow S(1,1)) = a B^2 \mp b B + c$. Hence, the pronounced minimum of $\Gamma (T_+ \rightarrow S(1,1))$ visible at $B \approx 0.65$ T results (mainly) from a combination of the negative linear and the positive quadratic contributions. The triplet-singlet transitions are several orders of magnitude slower compared to the rates shown in Fig. 3b. This is partly related to the fact, that the phonon spectral density decreases at low frequencies, and the energy differences between the S and T states are now relatively small. In addition, a strong delocalization of the electron states decreases some of the spin-orbit coupling mechanisms, that give rise to $T_\pm \rightarrow S(1,1)$ and $T_0 \rightarrow S(1,1)$ relaxation. On the other hand, the magnitude of $\Gamma (T_0 \rightarrow T_+)$ is very similar (with the difference of about 10%) to the case of $F=26$  kV/cm. This is due to the fact, that the $T_0 \rightarrow T_+$ transition involves spin-flips in the individual dots, which are not sensitive to the electric field.

### Two-phonon processes

As temperature grows, the role of two-phonon processes in spin relaxation increases, in particular between levels separated by a small energy difference^56–59^. In this section we calculate the two-phonon correction to the spin relaxation rates as a function of temperature and compare it to our results for single-phonon processes, presented in the preceding section. In addition, we calculate the singlet-triplet dephasing rate due to elastic phonon scattering involving virtual transitions to singlet states (2,0) and (0,2)^60,61^.

The time evolution of the reduced density matrix $\rho$ that describes the electron part of the system is determined (for a pure initial state) by the time-convolutionless (TCL) equation, which in the interaction picture can be written as^62^

$$\begin{aligned} \frac{d}{dt} \rho (t) = {{\mathscr {K}}}(t) \rho (t), \end{aligned}$$

where ${\mathscr {K}}(t)$ is a generator. Within the TCL projection operator technique^62^, the generator can be expanded up to a desired order in the coupling (here represented by the $H_{\mathrm {int}}$ part of the Hamiltonian). To simulate two-phonon processes, one needs to take into account terms up to the fourth order

$$\begin{aligned} {\mathscr {K}}(t) = \sum _{n}^4 K_n(t), \end{aligned}$$

where the explicit definitions of $K_n(t)$ are given e.g. in Refs.^60,62^. Note, that $K_4(t)$ describes not only two-phonon effects, but contains also perturbative corrections to the single-phonon processes. The relaxation rates from a state *i* to *j* are calculated within the Markov limit [$K_n(t \rightarrow \infty ) \equiv K^{(\infty )}_n$ ] by investigating the terms in the equation of motion for $\langle j | \rho | j\rangle$ that are proportional to $\langle i | \rho | i\rangle$. The rate includes the second-order contribution $\Gamma ^{(2)}(i \rightarrow j)$, corresponding to the single-phonon transition described in the previous Section. Performing calculations similar to those described in Refs.^60,61^, we further obtain the fourth-order contribution, accounting for the two-phonon processes

1 $$\begin{aligned} \Gamma ^{(4)}(i \rightarrow j)&= \; \pi \sum _{\alpha ,\beta } {\mathscr {P}} \int {\mathrm{d}}\omega \frac{1}{(\omega - \omega _{i \alpha })(\omega - \omega _{j\beta })} \nonumber \\&\quad \times {\mathrm{Re}} \Big [ R_{i \alpha j \beta }(\omega ) R_{\alpha j \beta i }(\omega _{ji} - \omega ) \nonumber \\&\quad + R_{i \alpha \beta i}(-\omega ) R_{\alpha j j \beta }(\omega _{ji} + \omega ) \Big ], \end{aligned}$$

where ${\mathscr {P}}$ denotes the Cauchy principal value, $\omega _{ij} = \left( E_j - E_i\right) /\hbar$, and the spectral densities are given by

$$\begin{aligned} R_{ijkl}(\omega )&= \frac{1}{\hbar ^2} \left| n_{\mathrm {B}}(\omega ) + 1\right| \sum _{\varvec{k},\lambda } G_{ij,\lambda }(\varvec{k}) G^*_{lk,\lambda }(\varvec{k}) [ \delta (\omega - \omega _{k,\lambda }) + \delta (\omega + \omega _{k,\lambda })]. \end{aligned}$$

In Eq. (1) the indices *i*, *j* refer to the singlet-triplet doublet of single-occupation states around the “sweet spot”, while $\alpha ,\beta$ run through all the states except to *i* and *j*. In the derivation of this result, we have selected only terms corresponding to energy-conserving two-phonon transitions between the two states, neglecting contributions that do not have a resonant two-phonon structure.

**Figure 5:**
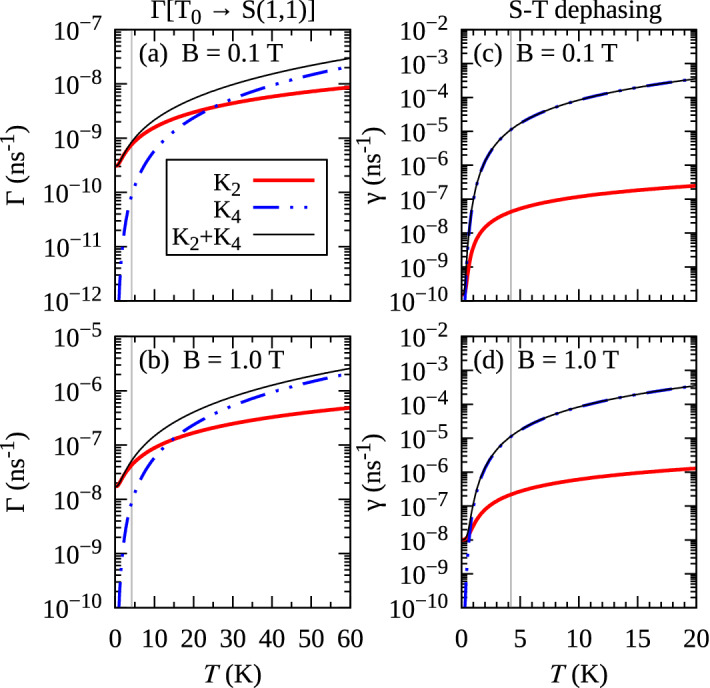
Temperature dependence of the phonon-assisted relaxation rate $\Gamma [T_0 \rightarrow S(1,1)]$ (**a**,**b**), and dephasing rate for the coherence represented by $\langle {S(1,1)}\vert{\rho }\vert{T_0}\rangle$ density matrix element (**c**,**d**). The electric field is taken at the “sweet spot” $F=14.87$ kV/cm. The vertical lines denote $T = 4.2$ K. Lines show the second- and fourth-order contributions, as well as the total rate, as defined in panel (**a**).

To assess the importance of the contributions coming from the two-phonon processes, we calculated the transition rates for two regimes of parameters. Figure 5a,b presents the temperature dependence of $\Gamma ^{(2)}(T_0 \rightarrow S(1,1))$ and $\Gamma ^{(4)}(T_0 \rightarrow S(1,1))$ for the electric field corresponding to the “sweet spot” $F=14.87$ kV/cm. While for low temperatures the single-phonon processes are much faster (by several orders of magnitude), the $\Gamma ^{(4)}(T_0 \rightarrow S(1,1))$ starts to dominate from $T \approx 25$ K (at $B = 0.1$ T) or $T \approx 14$ K (at $B = 1.0$ T). On the other hand, for the transitions $T_\pm \rightarrow S(1,1)$ (not shown here) the single-phonon processes dominate in the whole temperature range. The relative importance of the two-phonon processes in the case of low energy splitting between the involved states is due to the super-ohmic character of the phonon spectral density that makes single-phonon transitions unfavorable at low phonon frequencies, while inelastic phonon scattering gains importance as the range of occupied phonon states enhances with temperature. We also performed calculations for the regime of tunneling, at $F=26$ kV/cm and $B=0.1$ T (not shown here). In that case, the two-phonon contribution is not significant (at $T=60$ K it is two orders of magnitude smaller than the single-phonon rate).

While singlet-triplet transitions rely on spin-orbit coupling and are therefore relatively slow, dephasing of singlet-triplet superpositions can also result from a two-phonon process that can be understood as elastic scattering of a phonon, during which the system undergoes a virtual transition to one of the doubly occupied singlet states and back^60,61^. Within the singlet-triplet doublet around the “sweet spot”, this process is strong only for the singlet, when it is spin-conserving, while for the triplet it is very inefficient. This leads to distinguishability between the two states and, in consequence, to pure dephasing of singlet-triplet superpositions. The decay of coherence between the *S*(1, 1) and $T_0$ states appears in the second order as a result of transitions between these two states, as well as thermally activated spin-conserving transitions from *S*(1, 1) to *S*(2, 0) and *S*(0, 2), which are exponentially suppressed at low temperatures. The corresponding dephasing rate $\gamma ^{(2)}$, extracted from the $K_{2}$ term in the TCL expansion in the Markov limit, has the form (using short-hand indexing ‘1’ and ’ 3’ for the states *S*(1, 1) and $T_0$, respectively)

$$\begin{aligned} \gamma ^{(2)} = \pi \sum _{i} \big [ R_{1ii1}(\omega _{i1}) + R_{3ii3}(\omega _{i3}) \big ], \end{aligned}$$

which is consistent with the usual Lindblad equation obtained via Born-Markov approximation in the weak coupling limit^62^. To account for the fourth-order contribution, we use the approach from Ref.^61^ and extract the dephasing rate $\gamma ^{(4)}$ from the TCL generator $K_{4}$,

$$\begin{aligned} \gamma ^{(4)}&= \pi \sum _{\alpha ,\beta } \int _{-\infty }^{\infty } {\mathrm{d}}{\omega } {\mathrm {Re}} \left[ \frac{R_{1\alpha 1\beta }(\omega ) R^*_{1\beta 1\alpha }(-\omega ) }{(\omega +\omega _{1 \alpha })(\omega _{1 \beta } - \omega )} + \frac{R_{1\alpha \beta 1}(\omega ) R^*_{1\beta \alpha 1}(-\omega ) - R_{1\alpha \beta 1}(\omega _{1 \alpha }) R^*_{1\beta \alpha 1}(-\omega _{1 \beta }) }{(\omega -\omega _{1 \alpha })(\omega - \omega _{1 \beta })} \right] , \end{aligned}$$

where indices $\alpha$, $\beta$ run through all the states except to *S*(1, 1) and $T_0$. As shown in Fig. 5c,d, the dephasing is indeed much faster (a few orders of magnitude at the typical experimental temperatures about 4 K) than the relaxation between the two states. The two-phonon pure dephasing mechanism dominates also over the single-phonon contributions to dephasing, related to real transitions. This dephasing rate grows even more at electric fields closer to the resonances between the state *S*(1, 1) and *S*(2, 0) or *S*(0, 2)^63^.

### Relaxation kinetics

**Figure 6:**
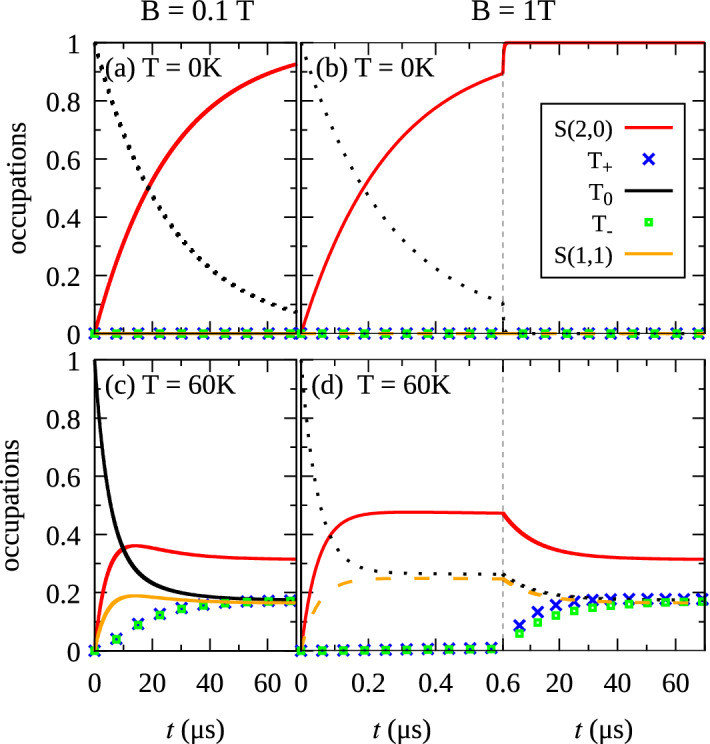
Time dependencies of the occupations at $F=26$ kV/cm. The vertical dashed lines divides two scales of the time axis.

To investigate the kinetics of the system, we numerically solve the Master equation (within the Markov and secular approximations) assuming the initial occupation of the $T_{0}$ state. We focus on the regime of the tunnel transitions, where we took $F=26$ kV/cm, and we consider magnetic fields of $B = 0.1$ T and $B = 1$ T. At $T=0$ K (Fig. 6a,b) the evolution is exponential, where the only significant process is the direct relaxation $T_{0} \rightarrow S(2,0)$. However, for a non-zero temperature, the picture becomes more complicated. The transitions to upper states become possible, and the final occupations form a temperature-dependent equilibrium (according to the Gibbs distribution). Furthermore, the Bose–Einstein distribution of phonons enhances the rates between states separated by a small energy difference. As shown in Fig. 6c,d, for $T=60$ K the evolution of occupations is no longer exponential and involves more states. The relaxation to the lowest singlet state can occur directly $T_{0} \rightarrow S(2,0)$, but also through $T_{0} \rightarrow S(1,1) \rightarrow S(2,0)$. Although the phonon-assisted transitions $T_{0} \leftrightarrow T_{\pm }$ are negligible, the states $T_{\pm }$ get occupied through $T_{0} \rightarrow S(1,1) \rightarrow T_{\pm }$, and $T_{0} \rightarrow S(2,0) \rightarrow T_{\pm }$ transitions. For a weak magnetic field (Fig. 6c), the occupation dynamics of all the triplet states exhibit comparable timescales. On the other hand, the higher magnetic field (Fig. 6d) leads to two distinct regimes: the fast transitions $T_{0} \rightarrow S(2,0)$ and $T_{0} \rightarrow S(1,1)$ in the first stage of the evolution, then slow transitions to the $T_\pm$. Such behavior results from different magnetic-field dependencies of the involved transition rates (see Fig. 3b).

**Figure 7:**
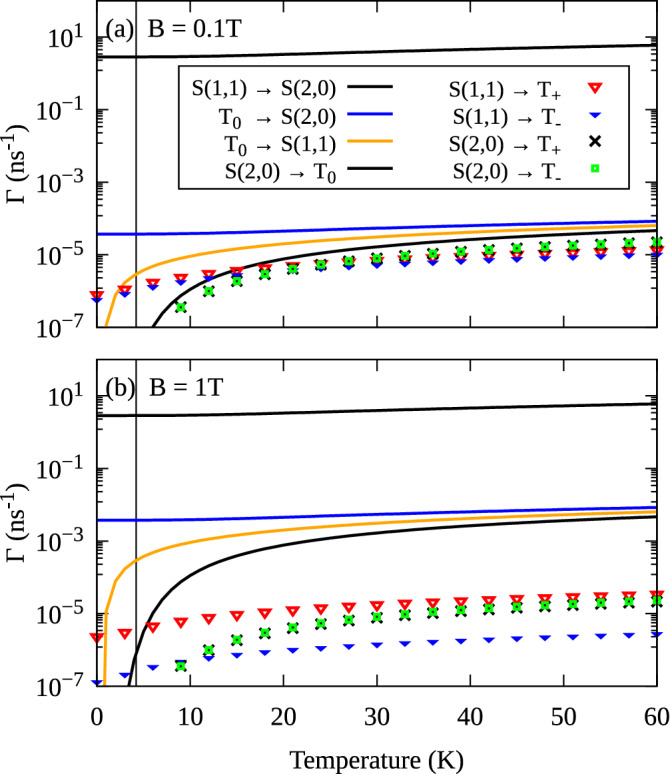
Temperature dependence of phonon-assisted transition rates at $F=26$ kV/cm. The vertical lines denote $T = 4.2$ K.

In order to quantitatively assess the importance of distinct transition channels for the kinetics presented in Fig. 6, we calculate the transition rates as a function of temperature (Fig. 7). For low temperatures, the relaxation is clearly dominated by the direct transition $T_{0} \rightarrow S(2,0)$. However, with increasing temperature and at low magnetic fields, the transitions involving the $T_\pm$ start to play an important role in the system dynamics. On the other hand, at higher magnetic fields ($B=1$ T in Fig. 7b), the transitions from/to the $T_{\pm }$ states are very slow compared to the $T_0 \rightarrow S(2,0)$ in the whole range of temperatures.

## Conclusions

We have studied quantum kinetics of two electrons in a quantum dot molecule. With a realistic model of the QD system geometry, 8-band $\varvec{k}\!\cdot \!\varvec{p}$ method, and configuration-interaction approach, we have calculated two-electron states. We have investigated the phonon-assisted transitions between the triplet and singlet states in the presence of external magnetic and electric fields. We have considered the triplet-singlet transitions accompanied by tunneling as well as the case of occupation conserving relaxation. We have identified channels of the triplet-singlet relaxation that become important in different parameter regimes. While for weak magnetic fields the tunnel transitions related to spin-admixture mechanisms are dominating, the regime of moderate and strong magnetic fields favors another mechanism related to the difference between the electron g-factors in the dots. We have also shown, that near the “sweet spot”, lifting of this mechanism leads to a considerably longer lifetime of the triplet $T_0$ state. We have also demonstrated a non-exponential quantum kinetics resulting from the interplay of various direct and sequential processes contributing to the relaxation. Finally, we studied the influence of two-phonon processes on the transition rates. We have shown that they can significantly contribute to the relaxation process at the “sweet spot”, at temperatures of several Kelvins and above. We have also confirmed that singlet-triplet coherence near the “sweet spot” is limited by the pure dephasing process due to elastic phonon scattering, which is much faster than relaxation.
